# Caprin-1 binding to the critical stress granule protein G3BP1 is influenced by pH

**DOI:** 10.1098/rsob.220369

**Published:** 2023-05-10

**Authors:** Tim Schulte, Marc D. Panas, Xiao Han, Lucy Williams, Nancy Kedersha, Jonas Simon Fleck, Timothy J. C. Tan, Xaquin Castro Dopico, Anders Olsson, Ainhoa Moliner Morro, Leo Hanke, Johan Nilvebrant, Kim Anh Giang, Per-Åke Nygren, Paul Anderson, Adnane Achour, Gerald M. McInerney

**Affiliations:** ^1^ Science for Life Laboratory, Department of Medicine Solna, Karolinska Institutet, and Division of Infectious Diseases, Karolinska University Hospital, Stockholm, 171 77, Sweden; ^2^ Department of Microbiology, Tumor and Cell Biology, Karolinska Institutet, Stockholm, 171 77, Sweden; ^3^ Division of Rheumatology, Immunity, and Inflammation, Brigham and Women's Hospital, Harvard Medical School, Boston, MA 02115, USA; ^4^ Harvard Medical School Initiative for RNA Medicine, Harvard Medical School, Boston, MA 02115, USA; ^5^ Protein Expression and Characterization, AlbaNova University Center, Royal Institute of Technology, 114 21, Stockholm; ^6^ Division of Protein Engineering, Department of Protein Science, School of Engineering Sciences in Chemistry, Biotechnology and Health, AlbaNova University Center, Royal Institute of Technology, 114 21, Stockholm; ^7^ Science for Life Laboratory, Tomtebodavägen 23A, 171 65, Sweden

**Keywords:** stress granule, G3BP, caprin-1, crystal structure, condensate, pH

## Abstract

G3BP is the central node within stress-induced protein–RNA interaction networks known as stress granules (SGs). The SG-associated proteins Caprin-1 and USP10 bind mutually exclusively to the NTF2 domain of G3BP1, promoting and inhibiting SG formation, respectively. Herein, we present the crystal structure of G3BP1-NTF2 in complex with a Caprin-1-derived short linear motif (SLiM). Caprin-1 interacts with His-31 and His-62 within a third NTF2-binding site outside those covered by USP10, as confirmed using biochemical and biophysical-binding assays. Nano-differential scanning fluorimetry revealed reduced thermal stability of G3BP1-NTF2 at acidic pH. This destabilization was counterbalanced significantly better by bound USP10 than Caprin-1. The G3BP1/USP10 complex immunoprecipated from human U2OS cells was more resistant to acidic buffer washes than G3BP1/Caprin-1. Acidification of cellular condensates by approximately 0.5 units relative to the cytosol was detected by ratiometric fluorescence analysis of pHluorin2 fused to G3BP1. Cells expressing a Caprin-1/FGDF chimera with higher G3BP1-binding affinity had reduced Caprin-1 levels and slightly reduced condensate sizes. This unexpected finding may suggest that binding of the USP10-derived SLiM to NTF2 reduces the propensity of G3BP1 to enter condensates.

## Graphical abstract

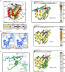

## 1. Introduction

Stress granules (SGs) are micron-sized membraneless compartments in eukaryotic cells that are dynamically induced upon environmental and biotic stresses such as oxidation and viral infections [1–3]. Dysregulated SGs are found in various diseases including cancer, viral infections and neurodegeneration [4–8]. SGs and other similar membraneless organelles are commonly referred to as biomolecular condensates (BMCs) [9]. BMCs result from liquid–liquid phase separation, a process that is governed by heterotypic multi-valent interactions between multi-domain proteins and nucleic acids [10]. The condensed protein state is characterized by increased inter-molecular order and may adopt distinct conformations with altered ligand binding [11–13]. Partitioning of BMC components follows basic thermodynamic principles and is dependent on changes in temperature and macromolecular concentrations, but also other less well-understood intracellular parameters such as pH or metabolite concentrations [10,14–16]. SGs contain mainly non-translating mRNAs and RNA-binding proteins as well as additional proteins affecting their functions [3,17–21]. The biomolecular composition and interaction networks of SGs are distinct from other BMCs, and also differ based on the type of applied stress or investigated cell type [17,18,22].

Three recent publications have elegantly demonstrated the key role of G3BP1 and G3BP2 (jointly referred to as G3BP) as effector molecules that mediate SG formation [23–25]. The authors combined systems biology, *in vitro* reconstitution systems, biochemical assays, cell biology and network theory to identify G3BP as the central hub and regulator of SG formation in selected eukaryotic cell lines. These studies not only confirmed numerous previous studies about the important role of G3BP in SG formation but also suggested a conceptual framework for G3BP-mediated condensation that is based on network theory [4,24,26–30]. According to this theory, macromolecules are defined, based on their number of interactions, as nodes (equal to or more than three interactions), bridges (two), caps (one) or bystanders (none). G3BP acts as a central node that possesses all the features required to drive phase separation and biomolecular condensation: homotypic oligomerization and heterotypic interactions mediated through the structural NTF2-like domain (hereafter referred to as NTF2), nucleic acid binding through the RRM and RGG domains as well as fine-tuning of SG formation through charged low complexity or intrinsically disordered regions (LCR/IDR) [19,23–25,31,32]. Caprin-1 and USP10 were identified as prominent regulators of G3BP-mediated condensation that promote and prevent SG formation, respectively [22,23,26–30]. Their disparate roles are explained in the described network theory which posits that USP10 acts as valence cap because it lacks both RNA-binding and oligomerization domains, but efficiently limits NTF2-mediated network interactions via its short linear FGDF motif [24]. Conversely, Caprin-1 acts as a bridge due to oligomerization and RNA-binding domains as well as its interaction with NTF2, via a short linear motif (SLiM) that is distinct from the biochemically and structurally well-defined FGDF motif [24,31,33,34].

G3BP-mediated SG condensation is triggered and regulated by electrostatic changes, but also other post-translational modifications such as ADP-ribosylation and phosphorylation [23–26,35–39]. Under non-stress conditions at neutral pH, G3BP1 adopts a phase separation inhibited compact state due to intra-molecular attraction between its acidic IDR and basic RGG domain, respectively. During stress, released mRNA binds to RGG, transforming G3BP1 into a phase-separation competent open state [25]. Furthermore, the phosphorylation status of the two serine residues Ser-149 and Ser-232 within the acidic region of G3BP1 may tune its threshold concentration for condensation [23–25]. A recent biophysical and functional *in vitro* deadenylation and translation study established that Caprin-1 and fragile X mental retardation protein-derived IDRs only phase-separated upon phosphorylation of either partner, and that the resulting phosphoprotein controlled deadenylation and translation activities due to differences in the nano-scale organization of the resulting condensates [35].

The pivotal role of NTF2 as an interaction hub is exemplified by studies demonstrating that Old World alphaviruses target NTF2 in order to recruit G3BP and associated 40S ribosomal subunits to the vicinity of viral cytopathic vacuoles (CPVs). For this purpose, Semliki Forest virus (SFV) and chikungunya virus express the non-structural protein 3 (nsP3) comprising a duplicated FGDF motif to outcompete USP10 for NTF2-binding [28,31,34]. The multi-domain protein nsP3 contains two FGDF motifs within a largely disordered hypervariable domain (HVD) for multi-valent protein interactions, and two structured domains including a macrodomain with ADP-ribosylhydrolase and eventual RNA-binding activities [34,40,41]. Strikingly, the recruitment of G3BP to CPVs via nsP3 results in the appearance of BMCs that are reminiscent of SGs but have translational activity [42].

In this work, we present the crystal structure of the central hub G3BP-NTF2 in complex with a Caprin-1-derived SLiM (PDB:6TA7), providing a molecular understanding of the mutually exclusive binding of USP10 and Caprin-1 to NTF2. The structure revealed two histidine residues on the surface of NTF2 in contact with Caprin-1 but not USP10. The discriminative contact was confirmed in biochemical and biophysical-binding assays. The size of stress-induced BMCs and *in vitro* condensates was modulated by substitution of the histidine residues to alanine or tyrosine. The interaction of USP10 with G3BP1-NTF2 was more stable and less sensitive to more acidic pH. Ratiometric fluorescence analysis revealed an approximately 0.5 pH unit drop in cellular condensates relative to the cytosol. Despite its higher binding affinity to NTF2, the expression of a Caprin-1/FGDF chimera led to reduced condensate sizes with reduced Caprin-1 levels. Thus, binding of the Caprin-1-derived SLiM to NTF2 may be more suitable than USP10 for the propensity of G3BP1 to enter condensates.

## 2. Results

### 2.1. Structural basis for mutually exclusive binding of USP10 and Caprin-1 to G3BP1-NTF2

**Figure 1.**
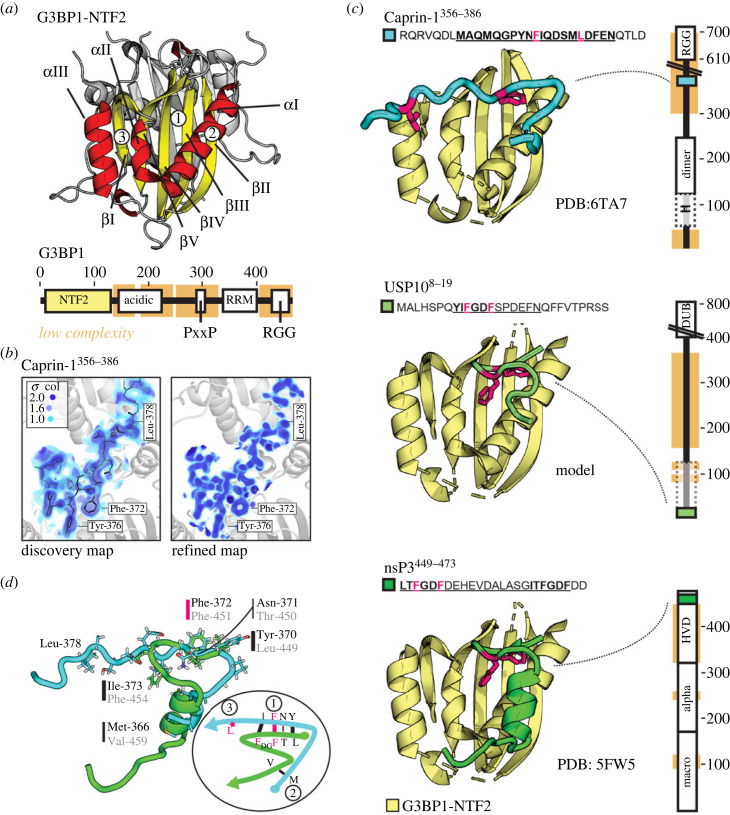
Mutually exclusive binding of USP10 and Caprin-1 to G3BP1-NTF2. (*a*) Helices and strands of G3BP1-NTF2 are shown in yellow and red. The second NTF2 subunit of the dimer is shown in grey, behind. Full-length G3BP1 (Uniprot: Q13283, scheme below) contains globular NTF2 and RRM domains, as well as structurally disordered regions, including a Glu-rich acidic region, an arginine–glycine–glycine (RGG) box RNA-binding motif and a Proline-rich SH3-binding motif (PxxP) [32]. The NTF2-like domain is in yellow and residue numbers are indicated above. (*b*) The initial 2mFo-DFc ‘discovery’ and refined maps of the Caprin-1^356–386^ fragment are visualized as volumes on depicted colour ramps. The Caprin-1^356–386^ model is shown in black within the blue density of the discovery map. (*c*) For simplicity, only helix and strand elements of NTF2 are drawn in yellow. Structural models of Caprin-1^356–386^ (PDB: 6TA7, crystal structure) and USP10^8–19^ (*in silico* homology model) as well as nsP3^449–473^ (PDB: 5FW5, crystal structure) are shown in cyan and green. The structural model of USP10^8–19^ was generated based on the high-sequence homology between its core (YI)FGDF motif and the (LT)FGDF motif of nsP3^449–473^ (as described in the electronic supplementary material, figure S2). Residues highlighted in pink were substituted to alanine to create G3BP1-NTF2 control mutants for ITC/BLI. Domain organizations: (top) human Caprin-1: N-terminal helical region (H), dimerization (dimer) and RNA-binding RGG domain (Uniprot: Q14444) [27,33,35]; (middle) USP10: deubiquitinase domain (DUB), N-terminal domain (dotted lines) that binds to p53 and contains PAM2 motif (Uniprot Q14694) [31,44,45]); (bottom) SFV nsP3: macro- and Zn-binding domains and a non-conserved HVD (Uniprot P08411) [34,40]). Low-complexity regions are shown in orange. (*d*) Structures of Caprin-1^356–386^ and nsP3^449–473^ are superimposed in cyan and green. The scheme highlights the residues of the shared binding regions of Caprin-1^356–386^ and nsP3^449–473^. The third binding region comprising Leu-378 is unique to Caprin-1^356–386^. For related details, see electronic supplementary material, figures S1 and S2*.*

Crystals obtained by co-crystallization of G3BP1-NTF2 with a synthesized peptide corresponding to residues 356–386 of Caprin-1 (Caprin-1^356–386^) diffracted to a maximum resolution of 1.9 Å, with diffraction spots of I/*σ* > 2 extending to 2.1 Å (electronic supplementary material, figure S1A). Six G3BP1-NTF2 molecules were identified in the asymmetric unit arranged as NTF2 dimers (electronic supplementary material, figure S1B). In agreement with previous studies [34,43], each NTF2 domain is composed of three α-helices *α*I–*α*III all lined up against a β-sheet created by the five β-strands *β*I–*β*V (figure 1*a*) showing minimal root-mean square deviations in the 0.3–0.6 Å range to the previous crystal structure. Larger structural deviations were only observed in loop regions. The initial discovery map revealed clear density for 20 of the 31 residues of Caprin-1^356–386^ that bound to one of the three NTF2 dimers (figure 1*b*). The final model was refined to *R* and *R*_free_ values of 20.8% and 25.2% (figure 1*b*; electronic supplementary material, figure S1C and table S1).

The comparison of the NTF2/Caprin-1^356–386^ and NTF2/nsP3^449–473^ crystal structures revealed that the 370-YNFI(Q)-374 segment of Caprin-1^356–386^ binds to the same hydrophobic G3BP1-NTF2-pocket (defined as site 1) that was previously identified for the (LT)FGDF motif of nsP3^449–473^ (figure 1*c*). Strikingly, the phenylalanine residue Phe-372 in Caprin-1^356–386^ adopts a conformation almost identical to that of residue Phe-451 in nsP3^449–473^ (figure 1*c,d*). Although Caprin-1^356–386^ and nsP3^449–473^ cover the same secondary NTF2-binding site (site 2), their N- and C-termini are aligned in opposite directions. Importantly, Caprin-1^356–386^ binding extends to a third NTF2-binding site (site 3) that is not covered by nsP3 (figure 1*c*,*d*). Due to its high-sequence homology with the (LT)FGDF motif of nsP3^449–473^, the (YI)FGDF motif of USP10^8–19^ is most probably positioned at sites 1 and 2, but not site 3, as visualized in a homology-based structural model (figure 1*c*; electronic supplementary material, figure S2A,B).

### 2.2. The entire length of Caprin-1^356–386^ binds G3BP1-NTF2 with low micromolar affinity

**Figure 2.**
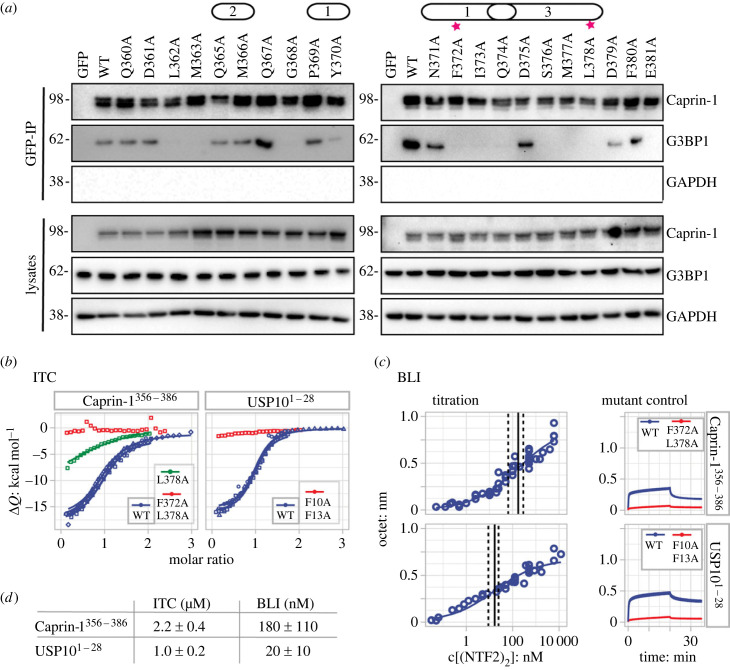
The entire length of Caprin-1^356–386^ binds G3BP1-NTF2 with low-micromolar affinity. (*a*) GFP-IPs of lysates were obtained from U2OS ΔCaprin-1 cells transiently expressing GFP-Caprin-1-WT and mutant constructs. G3BP1, Caprin-1 and GAPDH were detected by Western blot; cell lysates were applied as protein level controls. Pink stars indicate mutants used for biophysical measurements. Data are representative of at least four repeated experiments. Identified NTF2-binding sites 1, 2 and 3 are indicated above mutated positions. For related details, see electronic supplementary material*,* figure S3. (*b*) Integrated heats (data points) and globally fit-binding isotherms (lines) from ITC thermograms are plotted in panels for GFP-Caprin-1^356–386^ and GFP-USP10^1–28^. (*c*) Affinities were estimated from DR curves obtained by kinetic BLI measurements. Left panel: Responses were obtained as pseudo-equilibrium binding levels from the end of each association phase of the NTF2 concentration doses. GFP-Caprin-1^356–386^ and GFP-USP10^1–28^ data were combined from 32 and 40 binding curves. Right panel: in a separate set-up, kinetic binding curves demonstrated significantly lower NTF2 dimer (NTF2_2_)-binding to GFP-Caprin^356–386^-F372A/L378A and GFP-USP10^1–28^-F10AF13A (red traces) compared to WT (blue). Datasets were collected in sets of four comprising two WT as well as single mutant and ‘blank’ surfaces. (*d*) ITC- and BLI- derived *K*_D_ values for NTF2_2_-binding to GFP-Caprin-1^356–386^ and GFP-USP10^1–28^ are listed as means with s.d. For related details, see electronic supplementary material*,* figure S4.

To verify the binding interface revealed by the crystal structure, we performed alanine mutagenesis on residues Q360 to E381 in a full-length Caprin-1 construct expressed in U2OS cells lacking endogenous Caprin-1 (electronic supplementary material, figure S3). Caprin-1 mutant constructs were transiently expressed and immunoprecipitated to determine binding to G3BP1 (figure 2*a*). The results demonstrated that residues all along the Caprin-1 fragment were required for efficient binding, in particular site 1 contact-residues Tyr-370, Phe-372 and Ile-373, site 3 contact-residues Ser-376, Met-377, Leu-378, as well as site 1/site 3-contact-residue Gln-374. In addition, the site 1 associated but non-contact-residue Gly-368 was also critical for the interaction, probably conferring flexibility to the peptide backbone. It should be noted that substitution of Leu-362, Met-363, Asp-379 and Glu-381 also abolished or reduced binding, although these residues were not resolved or identified as contacting residues in the crystal structure. Ala-substitution of site 2 contact-residues Gln-365 and Met-366, site 1 contact-residues Pro-369 and Asn-371, site 3 contact-residues Asp-375 as well as of residues Gln-360, Asp-361 and Phe-380 did not affect binding and were indistinguishable from wild-type.

Previous studies revealed that Caprin-1 and USP10 compete for G3BP1-binding, suggesting proximal binding sites and similar binding affinities [2]. Indeed, isothermal titration calorimetry (ITC) experiments revealed similar low-micromolar affinities and thermodynamic signatures for both GFP-Caprin-1^356–386^ and GFP-USP10^1–28^ (figure 2*b*). Injection of GFP-Caprin-1^356–386^ to NTF2 yielded exothermic heat changes with initial peaks in the 0.5 µcal/s range that returned to baseline during the final injections (electronic supplementary material, figure S4A). The global heterogeneous 1 : 1 model fit to the derived binding isotherms from four GFP-Caprin-1^356–386^-NTF2 titrations yielded affinity (*K*_D_) and enthalpy (Δ*H*) values of 2.2 ± 0.4 µM and −16.4 ± 0.4 kcal mol^−1^ (figure 2*b*). Low-micromolar affinity and similar exothermic enthalpy values were also obtained for binding of GFP-USP10^1–28^ to NTF2 from three binding isotherms with *K*_D_ and Δ*H*-values of 1.0 ± 0.2 µM and −15.5 ± 0.4 kcal mol^−1^. Based on the co-immunoprecipitation (IP) assays and our previous studies [31,34], we selected GFP-Caprin-1^356–386^-F372A/L378A and GFP-USP10^1–28^-F10A/F13A as negative control mutants for ITC. Indeed, injection of these double mutants to NTF2 yielded minor heat fluctuations at baseline level (figure 2*b*; electronic supplementary material, figure S4A). Titration of single-mutated variant GFP-Caprin-1^356–386^-L378A caused heat changes that were due to an interaction with a significantly reduced affinity in the 20 µM range (figure 2*b*; electronic supplementary material, figure S4A). For complementary kinetic bio-layer interferometry (BLI) measurements, we measured NTF2 binding to GFP-USP10^1–28^/Caprin-1^356–386^ immobilized via chemical amino-coupling. Thereby, we determined apparent EC_50_-values for binding of NTF2 to GFP-USP10^1–28^ and GFP-Caprin^356–386^ with 20 ± 10 nM and 180 ± 110 nM, respectively (figure 2*c*; electronic supplementary material, figure S4B). This overestimated binding strength is caused by (i) rebinding of bivalent NTF2 dimers to the surface-immobilized ligands and (ii) electrostatic attraction of the basic NTF2 (pI of about 9) by the acidic sensor surface. Due to these limitations, as is often observed for not optimally designed biosensor-based assays [46], we did not interpret binding kinetics. However, specific binding of NTF2 was demonstrated by significantly reduced binding to the mutated controls GFP-Caprin-1^356–386^-F372A/L378A and GFP-USP10^1–28^-F10A/F13A (figure 2*c*; electronic supplementary material, figure S4B, right panels). Importantly, NTF2 bound stronger to USP10^1–28^ than to Caprin-1^356–386^ confirming the ITC-derived relative-binding strengths.

In conclusion, GFP-Caprin-1^356–386^ and GFP-USP10^1–28^ both bind G3BP1-NTF2 in a similar micromolar affinity range as determined by ITC, with slightly stronger binding by GFP-USP10^1–28^, observed by ITC and BLI.

### 2.3. Caprin-1 Leu-378 contacts G3BP1 His-31 and His-62 within a third NTF2-binding site beside those used by USP10^8–19^ or nsP3^449–473^

**Figure 3.**
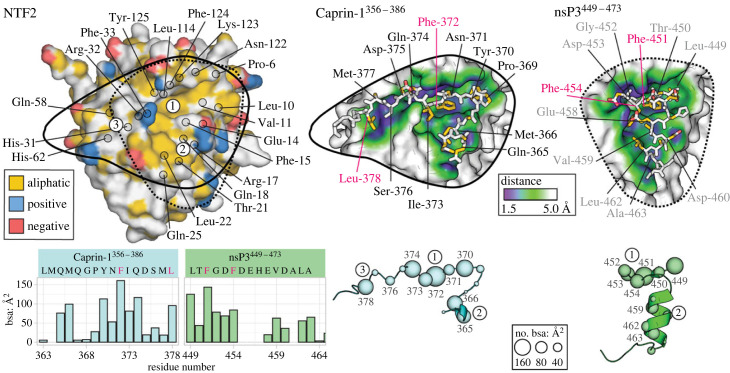
Caprin-1 Leu-378 contacts histidine residues His-31 and His-62 within a third NTF2-binding site outside those covered by USP10 or nsP3^449–473^. Top left: Functional aliphatic, negatively and positively charged groups are coloured yellow, red and blue on the NTF2 surface (yrb-coloured). Backbone-C_ɑ_ atoms of residues in contact with any of the two ligands are highlighted in black circles. Top right: Caprin-1^356–386^ and nsP3^449–473^ are visualized as yrb-coloured sticks on surface patches of NTF2 that are coloured according to the depicted distance ramp. The ramp visualizes closest distances of non-hydrogen atoms of NTF2 to those of the ligands. Bottom left: Residue-level surface areas (bsa) upon binding of Caprin-1^356–386^ and nsP3^449–473^ are visualized as bar-charts coloured in cyan and green, respectively. Bottom right*:* bsa of each residue of Caprin-1^356–386^ and nsP3^449–473^ is visualized in cyan and green on the depicted C_ɑ_ size scale. For related details, see electronic supplementary material*,* figure S2.

**Figure 4.**
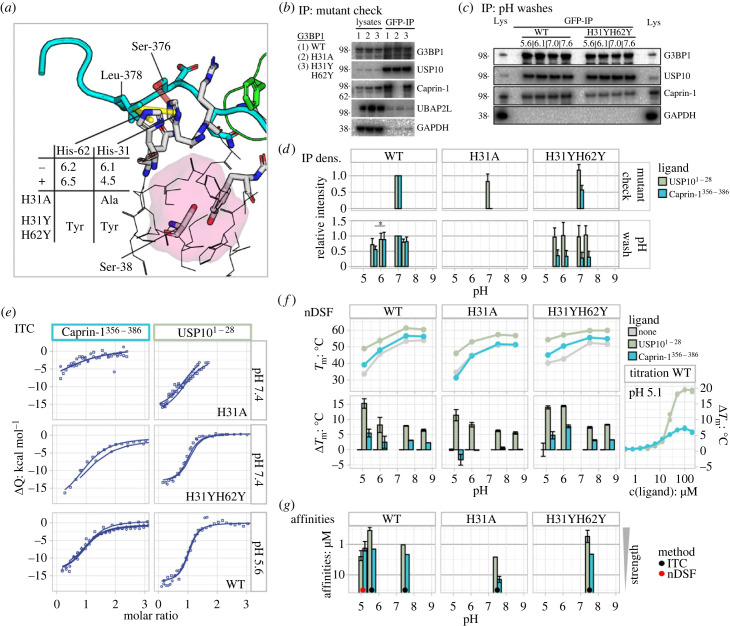
The NTF2 residue His-31 selects for Caprin-1 but not USP10. (*a*) Leu-378 is coordinated by His-31 and His-62 with predicted pK_a_-values of 6.1 and 6.2 as well as 4.5 and 6.5 in the unbound (−) as well as bound (+) states, respectively. Superimposed Caprin-1 and USP10 ligands are shown in cyan and green. Important side chains are highlighted as sticks. Ser-38 and interface residues exclusive to Caprin-1 binding are shown as grey sticks. Residues comprising atoms within a radius of below 8 and 4 Å from the Ser-38 side chain are shown as thin lines and highlighted by the semi-transparent red surface. Selected hydrophobic and hydrogen bond interactions are shown as yellow and red semi-transparent dashes. The two mutants described in the manuscript are highlighted. For related details, see electronic supplementary material, figure S5*.* (*b*) U2OS ΔΔG3BP1/2 cells were stably transfected with indicated GFP-G3BP1 mutant constructs. GFP fusions were precipitated using GFP-Trap agarose. Immunoprecipitates and full cell lysates were analysed by Western blot for G3BP1, Caprin-1, USP10 UBAP2L or GAPDH. (*c*) U2OS ΔΔG3BP1/2 cells were stably transfected with indicated GFP-G3BP1 mutant constructs. GFP fusions were precipitated using GFP-Trap agarose and washed with pH-adjusted buffers. Immunoprecipitates and full cell lysates were analysed by Western blot for G3BP1, Caprin-1, USP10 or GAPDH. (*d*) For comparative column plots, signals in each blot were quantified and normalized to the signal of wild-type G3BP1 at pH 7.0. Initial NTF2 mutant controls (upper panel) and pH washes (lower panel) were analysed separately. Data shown are mean ± s.e.m. and are analysed using an one-way ANOVA, *, *p* < 0.05; mutant control *n* = 5, pH washes *n* = 4. (*e*) ITC data of GFP-Caprin-1^356–386^ and GFP-USP10^1–28^ binding to H31A, H31YH62Y and WT at pH 7.4, 7.4 and 5.6 are visualized in figure 2*b*. For related details, see electronic supplementary material, figure S4*.* (*f*) In nanoDSF, G3BP1-NTF2 melting temperatures (*T*_m_) were detected as changes in Tyr/Phe fluorescence (*T*_m_-*F*) and derived from the data shown in the electronic supplementary material figure S5. Top panel: *T*_m_ values of G3BP1-NTF2-WT, -H31A and -H31YH62Y in apo-form (grey) as well as incubated with 10 x molar excess of USP10^1–28^ (green) and Caprin-1^356–386^ (cyan) peptides. Ligand-binding-induced stabilization (Δ*T*_m_) values were obtained by subtracting the *T*_m_ value of the apo- from ligand-bound forms. Titration data: peptides were titrated to G3BP1-NTF2 and affinity values were obtained by isothermal fitting [47,48]. For related details, see electronic supplementary material, figure S6*.* (*g*) Bar plot to compare affinity values from ITC (highlighted by black dots) and nanoDSF (red dot) on a logarithmic scale against pH. ITC and nanoDSF errors were obtained by F-statistics and the s.d. of values at four temperatures, respectively.

Analysis of residue-level buried surface areas highlighted Caprin-1^356–386^-YNFI(Q) and nsP3^449–473^-(LT)FGDF as the major interaction hot spots (figure 3; electronic supplementary material, figure S2C). The same major conclusions apply to the highly homologous (YI)FGDF motif of USP10^8–19^ (figure 1*c*; electronic supplementary material, figure S2A,B). Since both the nsP3-LTFGDF and Caprin-1-YNFI segments essentially cover the same NTF2-binding sites 1 and 2 (figures 1 and 3), we focused on site 3. This latter site 3, located between helices *α*II and *α*III, uniquely interacts with a stretch of five Caprin-1^356–386^ residues, Gln-374 to Leu-378 (figure 3). Caprin-1 residue Gln-374 (denoted as ^Caprin-1^Gln-374) is the connecting link between sites 1 and 3 and contacts NTF2 residues Phe-124, Tyr-125, Lys-123 and Arg-32. These interactions also comprise hydrogen bonds between the backbone amide groups of ^Caprin-1^Gln-374 and the backbone and side chain guanidine groups of ^NTF2^Arg-32, as well as another hydrogen bond between the side chain amide of ^Caprin-1^Gln-374 and the amino-group of ^NTF2^Lys-123 (figure 3). The backbone carbonyl-oxygen of ^Caprin-1^Asp-375 is fixed by a hydrogen bond to the side chain amide-nitrogen of ^NTF2^Gln-58 (residues are shown in figures 3 and 4*a*). Interestingly, the side chain of residue ^Caprin-1^Leu-378 is positioned centrally above the triangle that is created by the Cα atoms of NTF2 residues Gln-58, His-31 and His-62 (figures 3 and 4*a*). While the two pH-sensitive imidazole groups have *in silico* estimated pKa-values of 6.1/6.2 in the unbound state, Caprin-1^356–386^ binding shifts the pKa of ^NTF2^His-31 to a value of 4.5 due to hydrogen bond formation with ^Caprin-1^Ser-376, while the pKa of ^NTF2^His-62 is slightly increased to a value of 6.5 (figure 4*a*).

Co-IP assays confirmed the importance of these residues for binding of Caprin-1^356–386^ to site 3 of NTF2, since single-alanine substitutions of Caprin-1 residues Gln-374, Ser-376, Met-377 and Leu-378 abolished binding (figure 2*a*). It should be noted that GFP-Caprin-1^356–386^-L378A bound to NTF2 in our ITC measurements with a significantly reduced affinity of about 20 µM (figure 2*b*). Since histidine residues frequently act as acid–base components within catalytic reaction centres or as pH-sensitive interaction regulators [49–53], we applied a protein database (PDB) search strategy [54] to identify three-dimensional arrangements similar to the one created between NTF2 residues His-62 and His-31, as well as Caprin-1 residues Leu-378 and Ser-376. We located a motif comprising His-340, His-343, Met-336 and Ser-332 within the pH-sensitive polymerization site of fibrinogen (electronic supplementary material, figure S5A). The authors proposed a mechanism whereby ionization of either or both histidine residues determines the pH dependence of fibrin polymerization [55]. Since the NTF2 residues His-62 and His-31 are contacting residues of Caprin-1 but not USP10, we hypothesized that the interaction of NTF2 with Caprin-1, but not with USP10, might be affected by changes in local pH.

### 2.4. Reduced stability of NTF2 bound to Caprin-1 relative to USP10 at acidic pH

To investigate the importance of NTF2-site 3 for Caprin-1 binding, residues ^NTF2^His-31, His-62 and Gln-58 were mutated in the context of GFP-fused full-length G3BP1, expressed in U2OS ΔΔG3BP1/2 cells and investigated by IP. Substitution of ^NTF2^His-31 to alanine (or asparagine) abolished binding to Caprin-1, but hardly affected binding to USP10 (figure 4*b*; electronic supplementary material, figure S5B). Substitution of ^NTF2^His-62 to alanine markedly reduced binding, but substitution to asparagine did not (electronic supplementary material, figure S5B). Substitution of both ^NTF2^His-31 and His-62 to pH-independent aromatic tyrosine reduced binding to Caprin-1 by about 50%, while binding to USP10 was slightly increased (figure 4*b*). Substitution of ^NTF2^Gln-58 to glutamic acid abolished binding to Caprin-1 while binding to USP10 was unaffected (electronic supplementary material, figure S5B). ITC measurements at neutral pH revealed that GFP-Caprin-1^356–386^ bound to NTF2-H31A with a reduced affinity of 14 ± 10 µM while GFP-USP10^1–28^ bound to NTF2-H31A with an affinity in the 1–5 µM range, slightly higher than the value obtained for NTF2-WT (figure 4*e*; electronic supplementary material, figure S4A). In contrast with the reduced binding of GFP-Caprin-1^356–386^ to NTF2-H31YH62Y in IP assays (figure 4*b*), ITC revealed that both GFP-Caprin-1^356–386^ and -USP10^1–28^ bound to NTF2-H31YH62Y with affinity values close to or slightly below wild-type (figure 4*e*; electronic supplementary material, table S2). Taken together, these data demonstrate that G3BP1 residues His-31 and Gln-58 in site 3 are critical for the interaction with Caprin-1 while residue His-62 plays a minor role. Furthermore, G3BP1-H31YH62Y may be used as control in experiments to interrogate pH-dependent binding of Caprin-1 to NTF2.

Washes of immunoprecipitated G3BP1-wild-type complexes using buffers at selected pH values revealed slightly decreased levels of Caprin-1 relative to USP10 at pH 5.6 (figure 4*c*). The same protocol was applied on pre-formed ligand/G3BP1-H31YH62Y, revealing relatively constant levels of USP10 and Caprin-1 over the entire pH range. However, Caprin-1 levels were considerably reduced compared to G3BP1-WT (figure 4*c*). ITC measurements performed for G3BP1-WT at pH 5.6 yielded *K*_D_ and Δ*H* values of 0.4 ± 0.3 µM and −17.6 ± 1.7 kcal mol^−1^ for GFP-USP10^1–28^, as well as 1.4 ± 1.0 µM and −13.7 ± 2.3 kcal mol^−1^ for GFP-Caprin-1^356–386^, respectively (figure 4*e*; electronic supplementary material, figure S4A). Measurements at more acidic pH values were not performed due to NTF2 instability. Indeed, nano-differential scanning fluorimetry (nanoDSF) revealed reduced NTF2 stabilities at low pH, with melting temperatures (*T*_m_) dropping from 53.5 to 45.5 and 33.5°C upon shifting the pH from 7.4 to 6.1 and 5.1, respectively (figure 4*f*; electronic supplementary material, figure S6). Notably, the USP10^1–28^ peptide stabilized NTF2 significantly better than Caprin-1^356–386^ at the lower pH-values of 6.1 and 5.1, as the *T*_m_-values were increased by 8 and 15°C, compared to only 2.5 and 5.5°C for Caprin-1^356–386^. Similar ligand-binding-induced stabilization was observed for NTF2-H31YH62Y, although we noticed a larger difference for both ligands at pH 6.1, which may be due to an underestimated *T*_m_-value of the apo-form. Importantly, the absolute *T*_m_-values of apo- and ligand-bound forms of NTF2-H31YH62Y were increased by 6.5°C compared to wild-type NTF2 at pH 5.1. In control measurements, NTF2-H31A was stabilized by USP10^1–28^, but not Caprin-1^356–386^ (figure 4*f*). At neutral pH, the thermal stability of NTF2 was increased by 8 and 3°C when bound to USP10^1–28^ or Caprin-1^356–386^ (figure 4*f*; electronic supplementary material, figure S6). Isothermal analysis of thermal stability shifts [47,48] induced by binding of Caprin-1^356–386^ and USP10^1–28^ to NTF2-WT at pH 5.1 yielded similar affinity estimates for both ligands in the 1–5 µM range.

Thus, nanoDSF and IP data suggest an enhanced capacity of USP10^1–28^ but not Caprin-1^356–386^ to stabilize G3BP1-NTF2. Stabilization was more pronounced at acidic pH. Increased thermal stability of NTF2-H31YH62Y at low pH indicated a destabilizing effect of protonated histidine residues on wild-type NTF2.

### 2.5. Altered Caprin-1 binding to NTF2-site 3 mutants fine-tunes G3BP1-mediated condensation

**Figure 5.**
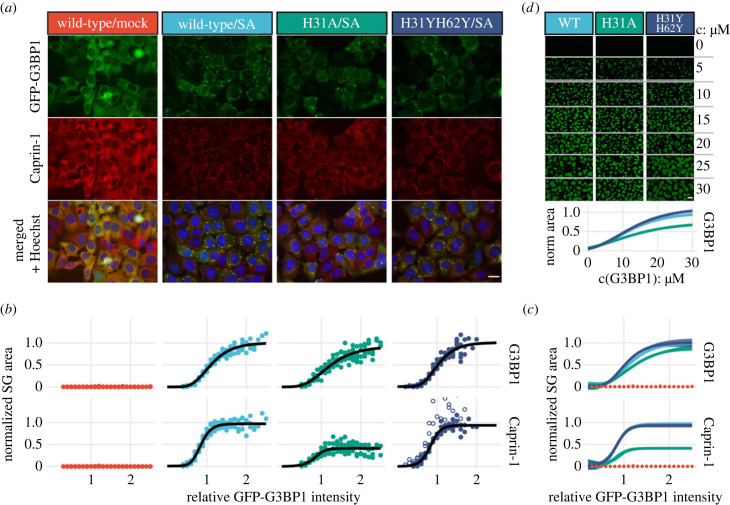
Altered Caprin-1 binding to NTF2-site-3 mutants fine-tunes G3BP1-mediated condensation. (*a*) Representative high-content microscopy images of U2OS ΔΔG3BP1/2 cells stably expressing G3BP1-WT, -H31A or -H31YH62Y were stressed with sodium arsenite (200 µM) for 1 h and thereafter compared to non-stressed (mock) cells. Cells were fixed and stained for Caprin-1 and Hoechst. Scale bar 20 µm. (*b*) The normalized SG area is plotted against the median GFP intensity value of cells grouped into intensity bins (relative GFP-G3BP1-intensity) in each experiment. SG areas were normalized to the maximal SG area of G3BP1-WT, separately for the GFP and Caprin-1 channels. For related details, see electronic supplementary material, figures S7 and S8*.* (*c*) Comparison of DR curves from (*b*) with added 95% confidence intervals*.* (*d*) The addition of recombinant G3BP1-WT, -H31A or -H31YH62Y to cell lysates from U2OS ΔΔG3BP1/2 cells stably expressing G3BP1-WT, -H31A or -H31YH62Y induced condensates of G3BP1 dependent on the dose of rG3BP1 increased in 5 µM steps. Images were taken 60 min after induction of condensate formation. DR curves with 95% confidence intervals were obtained as in (*b*). Scale bar 10 µm. For related details, see electronic supplementary material, figure S9*.*

Since Caprin-1 and USP10 bind G3BP in a mutually exclusive manner, promoting and impeding SG formation [2], we investigated SG formation in U2OS ΔΔG3BP1/2 cells stably expressing GFP-G3BP1-WT, -H31A or -H31YH62Y. Qualitative fluorescence microscopy images revealed that SG formation was impaired in G3BP1-H31A, but not in G3BP1-H31YH62Y, respectively (figure 5*a*). For a quantitative analysis by high-content imaging analysis GFP-positive cells of each G3BP1 construct were pre-sorted by fluorescence-activated cell sorting (FACS) on their GFP expression levels (electronic supplementary material, figure S7A). Our subsequent analysis of 6000–8000 cells expressing each construct revealed approximately 75% SG-positive cells upon SA treatment (electronic supplementary material, figure S7B). Although cells were pre-sorted by flow cytometry, relative GFP-G3BP1 expression levels were variable (figure 5*b*; electronic supplementary material, figure S7C), probably due to stochastic transcription and translation rates in individual cells [56]. Plots of SG sizes against GFP expression levels appeared as dose–response (DR) curves with varying maximal responses in each experiment (electronic supplementary material, figure S7D). After normalization of the data to the wild-type response, fitted DR curves reveal impaired condensate formation in H31A relative to WT and H31YH62Y (figure 5*b,c*). Specifically, the fitted maximal response and effective dose (ED50) values were reduced to 90% and increased by 0.2 relative fluorescence units (rfu) relative to wild-type in the GFP-G3BP1 channel. Interestingly, H31YH62Y cells exhibit slightly increased maximal response values and reduced ED50 values. DR curves of Caprin-1 labelled condensates revealed a significant loss of Caprin-1 in H31A cells, with a maximal response decreased by 50% relative to wild-type and H31YH62Y. The steeper DR curves for Caprin-1 recruitment into condensates may indicate limited Caprin-1 availability at higher GFP-G3BP1 concentrations. A similar impairment of Caprin-1 recruitment into condensates was observed for H31A and Q58E cells that were analysed in an earlier high-content imaging screen that was collected at a lower magnification, and for which cells were not sorted by FACS (electronic supplementary material, figure S8A). The cellular assays were complemented by high-fidelity condensate reconstitution according to established protocols [57]. Spiking of recombinant full-length wild-type, H31A or H31YH62Y G3BP1 into cell lysates yielded DR curves of condensate sizes in agreement with the cellular data. Condensate formation of H31A was impaired as revealed by a reduced maximal response of 70% and an increased ED50 by 5 µM. While DR curves of WT and H31YH62Y were similar, H31YH62Y appeared slightly shifted to lower ED50 and higher maximal response values, similar to the cellular condensation (figure 5*d*).

Thus, substitution of NTF2-His-31 to alanine decreased binding of Caprin-1 to G3BP1 and reduced its recruitment into granules. SG size in cells expressing GFP-G3BP1-H31A was reduced, in agreement with Caprin-1's previously demonstrated bridge function [2,23]. Sizes of *in vitro* reconstituted and cellular condensates of H31YH62Y cells were minimally increased relative to wild-type, suggesting a slight contribution of potentially protonated NTF2-His residues for SG formation assays.

### 2.6. In vitro cellular and reconstituted condensates are acidified by approximately 0.5 pH units, yet above the estimated pKa-values of the NTF2-site 3 histidine residues

**Figure 6.**
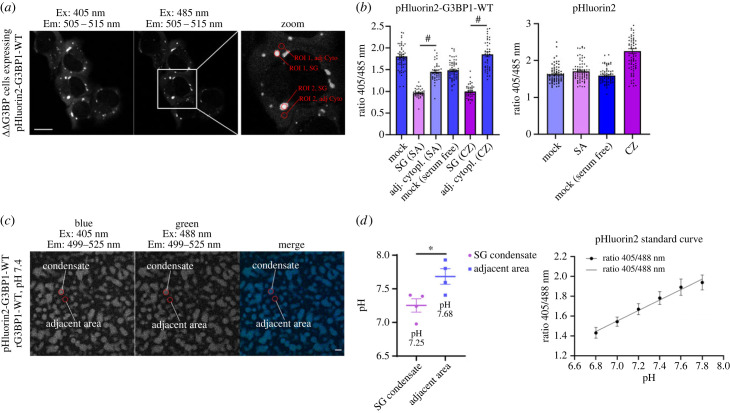
Lowered pH, detected in cellular SGs and reconstituted condensates. (*a*) pH-dependent ratiometric changes of pHluorin2 were monitored to estimate the intracellular pH within SGs and the adjacent cytoplasm. Fields of live-stressed U2OS ΔΔG3BP1//2 cells stably expressing pHluorin2-G3BP1-WT were sequentially excited at two excitation wavelengths of 405 and 485 nm, and the emission was detected between 505 and 515 nm. The enlarged image (zoom) exemplifies our procedure to derive fluorescence intensities from ROI within SGs and the adjacent cytoplasm. Scale bar 20 µm. (*b*) Images of U2OS ΔΔG3BP1/2 cells stably expressing pHluorin2-G3BP1-WT or pHluorin2 alone were analysed as described in (*a*), then 405/485 nm ratios were calculated and plotted. Data shown are mean ± s.e.m. and are analysed using an unpaired *t*-test. ^#^*p* < 0.001, *n* = 2. (*c*) Addition of recombinant G3BP1-WT (20 µM) to cell lysates from U2OS ΔΔG3BP1/2 cells stably expressing indicated pHluorin2-G3BP1-WT (pH 7.4) induces condensates. Condensates were excited at two excitation wavelengths of 405 and 488 nm, and the emission was detected between 499 and 525 nm. Then, we applied the same methodology to derive fluorescence intensities as shown in (*a*). Scale bar 10 µm. (*d*) Images of pHluorin2-G3BP1-WT condensates were analysed as described in (*a*), then 405/488 nm ratios were calculated and plotted. Data shown are mean ± s.e.m. and are analysed using an unpaired *t*-test. *p < 0.05, *n* = 4. pH adjusted pHluorin2 lysates were used for making a standard curve.

To estimate changes of pH in SGs relative to the adjacent cytoplasm, we measured pH-induced intensity changes of pHluorin2 [58] fused to G3BP1-WT in live U2OS ΔΔG3BP1/2 cells (figure 6*a*). The ratiometric GFP variant pHluorin2 exhibits a bimodal fluorescence excitation spectrum with peak maxima at 395 and 475 nm when the emission is recorded at the fluorescence peak maximum at 509 nm. Acidic pH decreases the fluorescence excitation peak at 395 nm, but increases at 475 nm, providing a means to monitor intracellular pH changes. Similarly performed analyses for the cytoplasm of stressed and non-stressed cells yielded fluorescence ratios of 1.5 and 1.8. Both sodium arsenite- and clotrimazole-induced SG (SG (SA) and SG (CZ)) had significantly reduced emission ratios with values of about 1, indicating considerably more acidic environments within these condensates relative to the adjacent cytoplasm (figure 6*b*). To further validate our findings from living cells, we performed an *in vitro* condensation assay with lysates from U2OS ΔΔG3BP1/2 cells expressing pHluorin2-G3BP1-WT and induced condensation with 20 µM rG3BP1-WT (figure 6*c*). Then, we analysed the emission spectra of condensates and adjacent areas as performed in figure 6*b* and, by using a pHluorin2 standard curve, we estimated a drop of pH of around 0.4 units from 7.7 to 7.3 in condensates (figure 6*d*).

To further test the influence of pH on condensation of SG proteins, we tested G3BP1-mediated condensation at different pH values. Condensates reconstituted at pH values between 6.8 and 7.8 revealed an optimum curve of condensate size with a maximum at pH 7.4 for G3BP1-WT (electronic supplementary material, figure S9). A similar optimum curve with reduced condensate sizes was recorded for G3BP1-H31A. Condensates of G3BP1-H31YH62Y cells were the largest over the entire pH range and continued to increase in size, up to pH 7.8. Reconstitution of condensates close to or below the pKa of histidine residues failed due to heavy precipitation of cell lysates. The observed differences are unlikely to be caused only by protonated site 3 histidine residues, but may be related to altered Caprin-1 binding and solubility changes of mutated G3BP1-NTF2.

### 2.7. Chimeric Caprin-1/FGDF yields smaller condensates despite higher binding affinity

**Figure 7.**
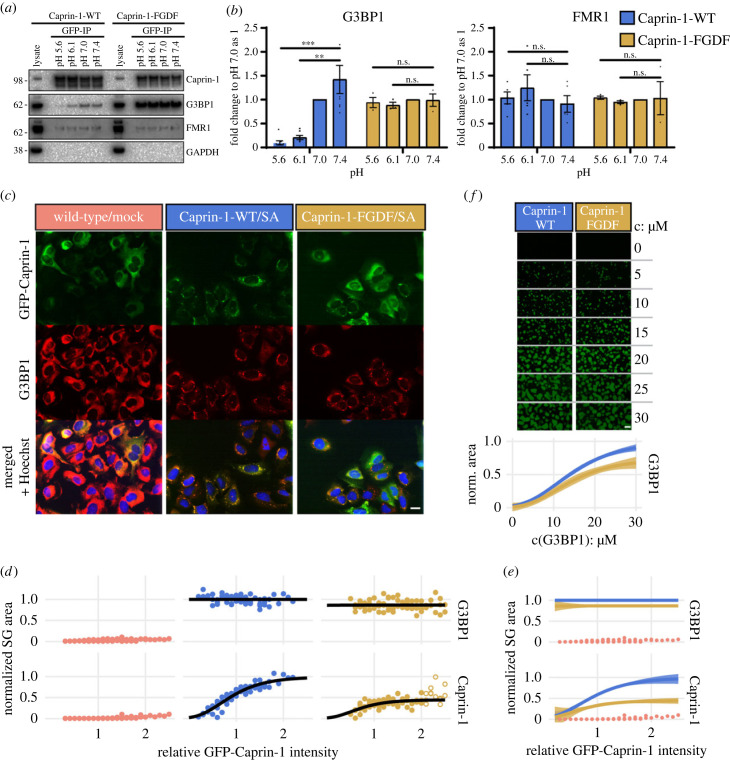
Chimeric Caprin-1/FGDF yields smaller condensates despite higher binding affinity. (*a*) U2OS ΔCaprin-1 cells were stably transfected with GFP-Caprin-1-WT or GFP-Caprin-1-FGDF. GFP fusions were precipitated using GFP-Trap agarose and washed with pH-adjusted buffers. Immunoprecipitates and full cell lysates were analysed by Western blot for Caprin-1, G3BP1, FMR1 or GAPDH. Data are representative of at least three repeated experiments. (*b*) Western blots signals were quantified by densitometry and the ratios of co-immunoprecipitated G3BP1 or FMR1 protein relative to GFP-Caprin-1-WT or GFP-Caprin-1-FGDF were determined. Data shown are mean ± s.e.m. and are analysed using an unpaired *t*-test; n.s.: non-significant, **p < 0.01; *n* = 8 for GFP-Caprin-1-WT and *n* = 3 for GFP-Caprin-1-FGDF IPs. (*c*) Representative high-content microscopy images of sodium arsenite (200 µM) stressed U2OS ΔCaprin-1 cells stably expressing GFP-Caprin-1-WT or GFP-Caprin-1-FGDF. Cells were fixed and stained for the indicated proteins. Scale bar 20 µm. (*d*) The normalized SG area is plotted against the median GFP intensity value of cells grouped into intensity bins (relative GFP-Caprin-1-intensity) in each experiment. SG areas were normalized to the maximal SG area of GFP-Caprin-1-WT, separately for the G3BP1 and GFP-Caprin-1 channels. For related details, see electronic supplementary material, figure S10. (*e*) Comparison of DR curves from (*d*) with added 95% confidence intervals. (*f*) The addition of 20 µM recombinant G3BP1-WT to cell lysates from U2OS ΔCaprin-1 cells stably expressing GFP-Caprin-1-WT or GFP-Caprin-1-FGDF induces condensates of GFP-Caprin-1 in a dose-dependent manner when concentration of rG3BP1-WT is increased by 5 µM steps. Images were taken 60 min after induction of condensate formation. DR curves with 95% confidence intervals were obtained as in (*d*). Scale bar 10 µm.

According to the network condensation theory, we expected increased condensation for a chimeric Caprin-1 construct in which residues 360–383 of Caprin-1 were substituted with residues 1–25 of USP10, including the FGDF motif (Caprin-1-FGDF) due to the increased affinity of this motif for G3BP1 and the loss of binding to site 3. Indeed, co-IP assays of lysates from FACS-sorted U2OS ΔCaprin-1 cells stably expressing GFP-Caprin-1-WT or -FGDF confirmed the higher binding affinity. Immunoprecipitated chimera levels were significantly higher than wild-type and also remained stable after acidic buffer washes (figure 7*a*,*b*). The interaction of the Caprin-1-binding protein FMR1 [35,39,59] was weakly detected but stable to changes in pH (figure 7*b*).

Despite its strong interaction with G3BP1, Caprin-1-FGDF was less efficiently recruited to SGs than Caprin-1-WT (figure 7*c*). This observation was confirmed by analysis of high-content imaging data obtained from FACS-sorted GFP-Caprin-1-WT or -FGDF-positive cells. Similarly to GFP-G3BP1 cells (figure 5*a*), variable GFP-Caprin-1 expression levels were detected. However, plots of SG sizes against GFP expression levels revealed DR curves only for Caprin-1, but not for G3BP1 (figure 7*d*,*e*; electronic supplementary material, figure S10). G3BP1 condensate size remained constant with increasing GFP-Caprin-1 levels, but was reduced by approximately 10% in GFP-Caprin-1-FGDF cells. Fitted DR curves of Caprin-1-specific condensates revealed a 50% size-reduction of Caprin-1-FGDF relative to wild-type cells (figure 7*d*,*e*; electronic supplementary material, figure S10). DR curves of *in vitro* condensate formation revealed a 30% reduced maximum size of condensates reconstituted in GFP-Caprin-1-FGDF lysates (figure 7*f*).

Thus, Caprin-1 levels below fluorescence detection level were sufficient to saturate the size of G3BP1-mediated condensates. Cells expressing GFP-Caprin-1-FGDF were impaired in granule formation and yielded reduced sizes of *in vitro* reconstituted condensates. These unexpected observations suggest that FGDF binding to the NTF2 domain can reduce the capacity of G3BP1 to transition into condensates.

## 3. Discussion

The crystal structure of the NTF2/Caprin-1^356–386^ complex at 2.1 Å resolution provides a molecular explanation for the mutually exclusive binding of Caprin-1 and USP10 to G3BP1-NTF2 [2,23–25]. The Caprin-1^356–386^-derived central-binding motif YNFI(Q) occupies the same hydrophobic-binding pocket on the NTF2 surface as do the USP10- and alphavirus nsP3-derived (YI/LT)FGDF motifs [34,43]. Both the Caprin-1 and USP10 /nsP3-derived SLiMs share a central phenylalanine that serves as the main aromatic anchor residue, adopting the same conformation in both ligands (figure 1). SLiMs are on average 6–7 amino acids in length and contain 3–4 core positions as main anchor residues, and bind their folded protein targets with affinities in the 1–500 µM range [60,61]. Homology-based structural modelling combined with biochemical data presented previously [2,24,31,34] and in this study suggest equivalent binding interfaces for the core (YI/LT)FGDF motifs of nsP3 and USP10 (figure 1; electronic supplementary material, table S2). Our conclusions have been recently confirmed by a study presenting crystal structures of both complexes [62]. While the nsP3^449–473^ and Caprin-1^356–386^-derived central LTFGDF and YNFI(Q) motifs cover interface surface areas close to 500 Å^2^ which is typically observed for SLiMs [60], the entire fragments cover interface areas of 740 and 860 Å^2^. Both USP10 and Caprin-1-derived SLiMs bind NTF2 with single-digit micromolar affinities as determined by ITC, placing both ligands at the higher affinity end of typical SLiM interactions.

Importantly, the crystal structure revealed that the Caprin-1^356–386^ residue Leu-378 contacts G3BP1-NTF2 residues His-31 and His-62, both localized within an extended-binding site (site 3) that is neither bound by nsP3 nor USP10. This discriminative contact is fixed by a hydrogen bond between the imidazole group of the NTF2 residue His-31 and the hydroxyl group of the Caprin-1 residue Ser-376. Notably, a structurally similar interaction was described for the pH-sensitive polymerization site of fibrinogen and proposed as a possible regulatory mechanism [55]. Our biophysical and biochemical assays demonstrated that alanine substitutions of NTF2-His-31 abolished binding to Caprin-1^356–386^ but only minimally affected the interaction with USP10. Further support for a ligand selection mechanism was previously provided by Sanders and co-workers who serendipitously discovered that a G3BP1-NTF2-S38F mutant binds USP10 but not Caprin-1 [24]. G3BP1-NTF2 Ser-38 is localized in the vicinity of site 3 (figure 4*a*), and we speculate that its substitution to a phenylalanine causes larger scale perturbations preventing Caprin-1 binding. Indeed, G3BP1-NTF2 Ser-38F accumulated in inclusion bodies when expressed in *E. coli* [63]. In line with the SG-network theory and recent phase-separation studies [23–25], disruptive mutations within the third NTF2-binding site reduced levels of Caprin-1 in condensates of stressed cells resulting in slightly smaller SGs. This was also confirmed in *in vitro* condensate assays (figure 5; electronic supplementary material, figures S7 and S8). Condensate size was dependent on GFP-G3BP1 expression levels, following apparent DR curves. However, reduced Caprin-1 recruitment into granules had only a minor effect on total granule size, in agreement with its previously demonstrated bridge function [2,23]. This minor effect on SGs is further supported by identification of a neurodevelopmental disorder associated Caprin-1-I373K mutation, causing only minor effects on SG/cell phenotype despite disrupted Caprin-1 recruitment [64]. It should also be noted that Caprin-1 is recruited into SGs independently of G3BP1 due to its interaction with mRNA and FMR1 [27,35,59]. The reduced SG condensation of the different mutants could also be attributed to more effective competition with USP10, which acts as a valency cap and blocks SG formation when overexpressed [2,23–25].

Identification of His-31 and His-62 residues in NTF2 in contact with Caprin-1 prompted us to test the pH dependence of this interaction. To estimate the pH in mammalian SGs, we fused the pH-sensitive GFP variant pHluorin2 to G3BP1-WT revealing a pH-drop of at least 0.5–1 units compared to the adjacent cytoplasm (figure 6*a*,*b*). This observation was supported by *in vitro* condensation assays that revealed a 0.5 unit reduction in condensates relative to the adjacent area (figure 6*c,d*). To render G3BP1-NTF2 site 3 less sensitive to changes in pH, residues His-31 and His-62 were substituted to tyrosine. Indeed, nanoDSF stability measurements revealed an increased stability of NTF2-H31YH62Y relative to NTF2-WT at acidic pH. Importantly, we observed an enhanced capacity of USP10 but not Caprin-1 to stabilize NTF2, especially at acidic pH (figure 4*f*). The different stabilization capacities could also be linked to the increased and reduced exothermic NTF2-binding enthalpies of USP10^1–25^ and Caprin-1^356–386^ at pH 5.6 in ITC (electronic supplementary material, table S2). However, ITC- and nanoDSF-derived affinity values for binding of USP10^1–25^ and Caprin-1^356–386^ to NTF2 at acidic pH were similar to the values obtained at neutral pH (figure 4*e–g*; electronic supplementary material, table S2). While both USP10 and Caprin-1 were washed away more easily from G3BP1-WT at acidic pH, this effect was less prominent for G3BP1-H31YH62Y, again indicating that histidine protonation destabilized G3BP1-NTF2-WT (figure 4*c*,*d*). In cellular SG and *in vitro* condensation assays, G3BP1-H31YH62Y cells exhibited a notable but non-significant condensate size increase, detected as slight left-shifted DR curves with an increased maximal response (figure 5). Condensates reconstituted *in vitro* from G3BP1-WT and G3BP1-H31A cell lysates at selected pH values between 6.8 and 7.8 revealed optimal curves of condensate sizes with a maximum size at pH 7.6, but overall larger condensates for G3BP1-WT. Notably, condensates obtained from G3BP1-H31YH62Y cells were the largest over the entire pH range, and in contrast with G3BP1-WT and G3BP1-H31A did not decrease at pH 7.8 (electronic supplementary material, figure S9). We note that the tested acidic pH of 6.8 is above the *in silico* estimated histidine pKa-values of 6.1/6.2 in the unbound state of NTF2. However, high RNA concentrations and a layered core–shell structure may reduce further the pH of nano-buffered compartments within SGs [65–68]. These data suggest that protonated His-31 and His-62 residues contribute to destabilize the NTF2 domain at low pH, which is compensated significantly better by USP10^1–25^ than by Caprin-1^356–386^ binding. Changes in pH rapidly and reversibly alter the electrostatic surfaces of interacting molecules, exemplifying a simple and evolutionarily conserved post-translational modification [69]. However, substitution of the two histidine residues to tyrosine did not significantly alter the size of SGs. Future analysis of SG assembly and disassembly using spatially and time-resolved imaging techniques may reveal yet hidden differential organization and dynamics of SGs in G3BP1-WT and G3BP1-H31YH62Y cells [67,70].

To examine the effect of the different binding modes of Caprin-1^356-386^ and USP10^1–25^ on the capacity of G3BP1-NTF2 to transition into SGs, we designed a chimeric Caprin-1-FGDF construct in which Caprin-1^360–383^ was exchanged for USP10^1–25^ (Caprin-1-FGDF). According to the posited condensation network theory [4,23–25], we expected increased condensate formation in cells expressing Caprin-1-FGDF due to the higher affine binding of the FGDF motif to NTF2. Co-IP assays confirmed the increased binding strength of Caprin-1-FGDF to G3BP1 (figure 7*a,b*). Notably, in cellular SG assays, Caprin-1 was recruited into granules following a DR relationship, while G3BP1 condensate size was independent of GFP-Caprin-1-FGDF or -WT concentrations. However, we cannot exclude differences in organization or stability of the granules with increased Caprin-1 concentrations. Strikingly, Caprin-1-FGDF was not as efficiently recruited into granules as Caprin-1 WT and granule size was reduced (figure 7*c*–*e*; electronic supplementary material, figure S10A). These data suggest that FGDF binding to the NTF2 domain reduces the propensity of G3BP1 to transition into the condensed SG phase. Such modulatory ligand behaviour has been referred to as polyphasic linkage, a framework to describe quantitatively how ligand-binding modulates the phase transition of scaffolds by preferential binding to their dilute and dense phases [12,13]. As outlined in the introduction, condensation-modulating ligands have been mainly defined by their valency, i.e. Caprin-1 was transformed from a bridging to a capping molecule by removal of its RNA-binding domain [13,24]. However, our data suggest valency-independent preferential binding of USP10^1–25^ to G3BP1 in the dilute phase. Such a preference may also provide an additional competitive advantage for Old World alphaviruses using a duplicated FGDF motif to recruit G3BP out of granules to the vicinity of viral CPVs [28,31,34,42].

Herein we have provided structural and biochemical evidence that in comparison to USP10^1–25^, Caprin-1^356-386^ selectively contacts two G3BP1-NTF2 histidine residues to form a complex that is less stable at acidic pH. Furthermore, we have discovered a more acidic pH in SGs, which may not only cause conformational changes of full-length G3BP1, but also modulate its interaction with ligand-derived SLiMs. Based on these and previous findings, we suggest the following mechanism to contribute to SG assembly and disassembly [4,23–25]: under normal conditions, Caprin-1 stably competes with other proteins such as USP10 and Ubap2/Ubap2L for site-specific binding to G3BP1-NTF2. Upon stress, Caprin-1/G3BP1 coalesces into SGs facilitated by multi-valent higher-order RNA interactions. In the acidic microenvironment of assembled SGs, the site-specific Caprin-1/G3BP1-NTF2 interaction is destabilized, providing a competitive advantage for USP10 binding. After stress release, USP10/G3BP1 transitions more rapidly out of the condensed into its preferred dilute phase, thus facilitating SG disassembly. The various roles of Caprin-1 in cancer as a cell cycle regulator and in neurodegenerative disease as potential contributor to altered RNA metabolism and also the roles of G3BP in viral infections, cancer and neurodegenerative disease highlight the importance of this interaction. Our work thus uncovers new targets for the potential treatment of diseases characterized by dysregulation of SGs [7,14,64,71–73].

## 4. Materials and methods

### 4.1. Heterologous protein production

G3BP1-NTF2 (residues 1–139, List S1) was expressed in *E. coli* and purified as previously described [34]. Briefly, the G3BP1-NTF2 dimer was affinity-purified (immobilized metal affinity chromatography (IMAC), HisTrap FF, GE Healthcare) and isolated from a Superdex 75 size exclusion chromatography (S75, SEC, GE Healthcare). After TEV cleavage (PSF, KI, Stockholm), G3BP1-NTF2 was collected as IMAC flow-through and re-applied on the same S75 column. For crystallization, the Caprin-1-derived peptide comprising residues 356–386 (RQRVQDLMAQMQGPYNFIQDSMLDFENQTLD) was dissolved in dH2O and added to G3BP1-NTF2 in five times molar excess. The G3BP1-NTF2/Caprin-1^356–386^ mix was dialysed against 20 mM HEPES, 300 mM NaCl, 10% glycerol, 1 mM TCEP, pH 7.5 (HEPES buffer TCEP) and concentrated to a total absorbance of 8.5 using ultrafiltration with a MW cutoff of 3 kDa. DNA fragments encoding Caprin-1^356–386^ and USP10^1–28^ were ligated to the 3′ end of fragment encoding GFP containing N-terminal twin-Strep-tag-II and TEV cleavage sites on pET21d-expression vectors (in-house, GFP-USP10^1–28^ and GFP-Caprin-1^356–386^). These modified vectors and expression constructs (List S1) were obtained by sequence and ligation-independent cloning and validated by DNA sequencing (Eurofins Genomics) [74]. GFP-USP10^1–28^ and GFP-Caprin-1^356–386^ were expressed in *E. coli* BL21 T7 Express. Bacterial pellets were lysed in HEPES buffer comprising 0.4 g l^−1^ lysozyme, 0.05 g l^−1^ DNase, 1 mM PMSF and 1 tablet/50 ml Roche EDTA-free protease inhibitor cocktail by a mechanical pressure cell (Homogenising Systems). STII-GFP-USP10^1–28^ and STII-GFP-Caprin-1^356–386^ were affinity-purified using Strep-Tactin columns (IBA). Tags were cleaved by incubation with TEV protease in HEPES buffer containing 1 mM EDTA and 1 mM DTT. Cleaved proteins were isolated from a Superdex 75 column (GE Healthcare) equilibrated in ITC-buffer (25 mM HEPES, 150 mM NaCl, 10 mM MgCl_2_, 10% glycerol, pH 7.5).

### 4.2. Crystal structure determination

Crystals were refined around the initial hit condition comprising 0.1 M Tris pH 8.0, 0.2 M NaCl, 20% (w/V) PEG 4000 from the Proplex crystallization screen (Molecular Dimensions) and set-up in 96-well sitting drop iQ plates using the Mosquito LCP robot (TTP Labtech). Crystals were cryo-protected by soaking in mother liquor supplemented with 30% (w/V) glucose and flash-frozen in liquid nitrogen. X-ray data were collected at beamline BL14-1 at the BESSY synchrotron radiation facility (Berlin, Germany) [75]. Diffraction data were collected to a resolution of about 1.9 Å and processed using the XDS/XDSAPP program package [76,77] (electronic supplementary material, figure S1A and table S1). The molecular replacement software Phaser placed four NTF2 monomers into the asymmetric unit (asu) that were manually extended to comprise six NTF2 molecules as evident from the electron density in Coot (electronic supplementary material, figure S1B) [78,79]. The *R* and *R*_free_ values of the refined manually extended MR solution were 28.6 and 34%. The electron density clearly revealed additional density (discovery map) to build 20 residues of the co-crystallized 31-AA long Caprin-1^356–386^ peptide bound to NTF2 chain D. An additional four residue short fragment comprising a phenylalanine residue was modelled as bound to NTF2 chain F, and the final model was refined further to *R* and *R*_free_ values of 20.8 and 25.2%. Coot and Phenix were used to manually re-build and automatically refine the model, comprising non-crystallographic restraints, individual isotropic B-factors and automatically determined TLS groups [78,80]. Residue-level map-to-model correlation plots were obtained using Phenix and R tidyverse [80,81]. Structural analysis was performed using PDBePISA, PyMol, Coot, PIC and ROSIE web servers [54,78,82–87]. Residue-level interaction summary plots and associated PyMol scripts were generated using *R* and the tidyverse package to analyse interaction tables obtained from Molprobity [81,88–90].

### 4.3. Biophysical interaction assays

#### 4.3.1. Isothermal titration calorimetry

Buffers of GFP-USP10^1–28^ and GFP-Caprin-1^356–386^ as well as G3BP1-NTF2 were exchanged to ITC-buffer (25 mM HEPES, 150 mM NaCl, 10 mM MgCl_2_, 10% glycerol, pH 7.5) by S75-SEC. ITC measurements were performed using an ITC200 calorimeter (GE Healthcare). The other buffers used to collect biophysical ITC or nanoDSF data at different pH-values were 25 mM MES pH 6.1 or pH 5.6 as well as 25 mM Na-acetate pH 5.1, 150 mM NaCl, 10 mM MgCl_2_, 10% glycerol (V/V). The cell temperature was set to 25°C and the syringe stirring speed to 750–1000 rpm. Before each experiment, G3BP1-NTF2 and GFP-USP10^1–28^ or GFP-Caprin-1^356–386^ were loaded into the cell and syringe at concentrations of 10–25 µM and 150–500 µM, respectively. Data and binding parameters were pre- analysed using the MicroCal PeakITC software (Malvern). Final analysis and global fits were performed using NITPIC and Sedphat [91,92].

#### 4.3.2. Prometheus nano-differential scanning fluorimetry

Prometheus nanoDSF measurements were performed in duplicate by loading NTF2 samples at absorbance values of 0.2 into high-sensitivity capillaries following the manufacturer's protocols (Nanotemper). Peptide ligands were dissolved in DMSO and added to NTF2 to yield final DMSO concentrations of 4% (V/V). The DMSO concentration was kept constant at 4% during the titration experiments. Buffers were identical to the ITC measurements. All data were combined, tidied and visualized applying ggplot2 and the tidyverse packages in Rstudio [81,89,90].

#### 4.3.3. Bio-layer interferometry

All BLI measurements were performed on an Octet RED instrument (ForteBio). GFP-USP10^1–28^ and GFP-Caprin-1^356–386^ proteins were immobilized covalently on the surface of Amine Reactive Second-Generation (AR2G) Biosensors following the manufacturer's protocols (ForteBio). Surfaces were re-generated applying cycles comprising three 5 s dips in 10 mM HCl and HEPES buffer after and before each titration experiment. For the titration experiments, four of the eight sensors in each sensor set were used as a reference and treated only with sulfo-NHS/EDC and ethanolamine, but not loaded with GFP-USP10^1–28^ and GFP-Caprin-1^356–386^. The stability of the sensor surfaces was monitored by including 500 nM NTF2_2_ samples in each experiment. In a second set of comparative experiments, GFP-USP10^1–28^ and GFP-Caprin-1^356–386^ WT and mutant proteins were loaded on two and one sensor surfaces, respectively. One of the remaining sensors was used as blank, and the other as an additional GFP-USP10^1–28^ WT surface. In this second set of experiments, NTF2_2_ was applied at the same concentration for all sensors and measured in parallel. Data were collected in 25/25 mM HEPES/MOPS pH 7.4, 1% glycerol (V/V), 0.05% Tween-20 (V/V), 150 mM NaCl. Raw data were pre-processed by subtracting the reference from the sample surfaces and applying a Savitzky-Golay filter as implemented in the Octet RED analysis software.

### 4.4. Cell lines and transient transfections

All cell lines were maintained at 5.0% CO_2_ in DMEM containing 10% fetal bovine serum, 100 U ml^−1^ penicillin and 100 µg ml^−1^ streptomycin. U2OS ΔΔG3BP1/2 stably expressing GFP-G3BP1-WT, H31A, H31YH62Y, pHluorin2 or pHluorin2-G3BP1-WT, or U2OS ΔCaprin-1 cells stably expressing GFP-Caprin-1-WT or FGDF chimera were made as described in detail elsewhere [93]. Cells were grown to 70–80% confluency and transfected using Lipofectamine 2000 (Invitrogen) following the manufacturer's instructions. Then selected with 100 µg ml^−1^ G418 (Gibco) for 7–10 days and screened using fluorescence microscopy and Western blotting. For transient transfections, cells were grown to 70–80% confluency and transfected using Lipofectamine 2000 (Invitrogen) following the manufacturer's instructions and processed after 24 h.

### 4.5. Applying CRISPR-Cas9 to obtain ΔCaprin-1 cell line

U2OS-WT (ATCC Cat# HTB-96, RRID:CVCL_0042) cells were plated and transfected with the pCas9-Guide (Origene GE100002, electronic supplementary material, table S3) constructs using Lipofectamine 2000 overnight, allowed to recover for greater than or equal to 2 days, and thereafter were reseeded and stained for Caprin-1 and G3BP1. Cultures with less than 5% KO cells were first ‘pool cloned’ to enrich for KOs by plating at 5–10 cells per well in 24-well plates, allowing the cells to grow to greater than 50% confluency before reseeding on coverslips in a 24-well plate for screening. When the cells on coverslips reached 80% confluency, cells were fixed and stained for Caprin-1 and G3BP1. Samples showing desired KO greater than 5% were subcloned by limiting dilution. To identify Cas9-induced mutations in the Caprin-1 coding sequence, genomic DNA was isolated using Trizol reagent (Thermo Fisher). Amplification of the genomic DNA was performed using a primer set (electronic supplementary material, table S4) spanning Exon 2 of genomic Caprin-1. Genomic DNA PCR was performed with DreamTaq PCR Master Mix (Thermo Fisher). DNA was initially denatured at 95°C for 1 min, followed by denaturation at 95°C for 30 s, annealing at 60°C for 30 s and extension at 72°C for 1 min for 30 cycles. Final extension was done at 72°C for 5 min. PCR products were gel purified and directly cloned into pCR.2.1 vector (Thermo Fisher). Individual clones were propagated, plasmids isolated and sequenced.

### 4.6. Cell sorting

U2OS ΔΔG3BP1/2 stably expressing GFP-G3BP1-WT, H31A, H31YH62Y, pHluorin2 or pHluorin2-G3BP1-WT, or U2OS ΔCaprin-1 cells stably expressing GFP-Caprin-1-WT or FGDF chimera were expanded in a 75 cm^2^ flask until confluent. Cells were then washed with PBS, trypsinized, collected in a 50 ml tube, washed with 40 ml PBS, resuspended in 3 ml FACS buffer (PBS, 0.5% FBS, 0.05% sodium azide, 5 mM EDTA) and sorted for GFP signal on a BD FACS Aria Fusion instrument. Parental U2OS ΔΔG3BP1/2 or U2OS ΔCaprin-1 cells were used as negative controls to determine the background signal in the FITC channel. Approximately 1.0 × 10^5^ cells per cell line with GFP fluorescence intensity between 10^4^ and 10^5^ were sorted, reseeded in 75 cm^2^ flasks and cultivated until confluent.

### 4.7. Molecular cloning

#### 4.7.1. Creation of pEGFP-G3BP1 and pAc-GFP-Caprin-1 mutants

The 5′ phosphorylated primers (electronic supplementary material, table S4) were mixed with 20 ng of pEGFP-C1-G3BP1-WT or pAc-GFP-C1-Caprin-1-WT according to protocol with Phusion DNA polymerase (Thermo Fisher) at a final volume of 20 µl. The mixture was denatured at 98°C for 45 s, followed by 28 cycles of the following: 98°C for 15 s, 61–66°C (depending on primer set) for 15 s, 72°C for 4 min, with a final extension step of 72°C for 12 min. The PCR mixture was incubated with one-unit DpnI (NEB) for 1 h at 37°C in a final volume of 40 µL to remove *E. coli*-derived template DNA, followed by heat inactivation at 80°C for 20 min. Fifty nanograms of the PCR product was ligated with T4 DNA ligase (NEB) in a final volume of 10 µl overnight at 4°C. 5 µl of the ligation mix was used for chemical transformation into high-efficiency five alpha *E. coli* (NEB).

#### 4.7.2. Creation of pAc-GFP-Caprin-1Δ360–383-USP101–25 (GFP-Caprin-1-FGDF chimera)

This plasmid was created using NEBuilder HiFi DNA assembly protocol (NEB). In short, backbone pAc-GFP-C1-Caprin-1^Δ360–383^ was created via Phusion PCR protocol (see above) with primers (electronic supplementary material, table S4) containing a 20 nt overhang to the USP10^1–25^ insert. This USP10^1–25^ insert was also amplified with primers (electronic supplementary material, table S4) containing a 20 nt overhang to the pAc-GFP-C1-Caprin-1^Δ360–383^ backbone via a Phusion PCR protocol (see above) based on a pEGFP-C1-USP10^1–40^-WT template [31]. Both PCR products were mixed with NEBuilder HiFi DNA assembly master mix according to the manufacturer instructions and five alpha *E. coli* (NEB) were chemically transformed. Plasmids were isolated and sequenced.

### 4.8. Immunoprecipitation and immunoblotting

#### 4.8.1. Standard procedure

Sixty millimetres dishes of 70–80%-confluent of U2OS ΔCaprin-1 cells were transiently transfected for 24 h, washed with PBS, and scrape harvested at 4°C into EE lysis buffer (50 mM HEPES, 150 mM NaCl, 2.5 mM EGTA, 0.5% NP40, 10% glycerol, 5 mM EDTA, 1 mM DTT, HALT protease inhibitors (Thermo Fisher). U2OS ΔΔG3BP1/2 stably expressing GFP-G3BP1-WT and mutated versions (G418 selected) were grown in 6-well plates until 70–80% confluency washed with PBS and scrape harvested at 4°C into EE lysis buffer. Lysates were rotated for 10 min at 4°C, sonicated for 8 min on ice, cleared by centrifugation (10.000 g, 10 min, 4°C) and incubated with anti-GFP beads for 1 h with continuous rotation at 4°C. Anti-GFP beads were produced by expressing the GFP nanobody ‘Enhancer’ [94] in *E.coli*, purifying by size exclusion, and coupling to cyanogen bromide-activated sepharose (Sigma-Aldrich) or commercial GFP-Trap agarose (Chromotek). Beads were washed in EE lysis buffer and eluted directly into 2 x NuPAGE LDS sample buffer with 100 mM DTT and denatured for 5 min at 95°C. Proteins were resolved in 4–12% Bis-Tris NuPAGE gels (Thermo Fisher Scientific) and transferred to nitrocellulose membranes using Trans-Blot Turbo Transfer system (Bio-Rad) and blotted using standard procedures and antibodies listed in the electronic supplementary material, table S5. Chemiluminescence was detected using SuperSignal West Pico substrate (Thermo Fisher).

#### 4.8.2. Ph-dependent immunoprecipitation (G3BP1)

8 × 10^6^ U2OS ΔΔG3BP cells stably expressing GFP-G3BP-1-WT or GFP-G3BP-1-H31YH62Y (FACS sorted) were lysed in 1.2 ml (50 mM HEPES pH 7.0, 150 mM NaCl, 2.5 mM EGTA, 0.5% NP40, 10% glycerol, 1 mM DTT, HALT protease inhibitors (Thermo Fisher), 100 µg ml^−1^ Heparin. Lysates were rotated for 10 min at 4°C, sonicated for 8 min on ice, cleared by centrifugation (10 000 g, 10 min, 4°C) and incubated with GFP-Trap agarose beads (ChromoTek) for 1 h with continuous rotation at 4°C. Then, beads were pelleted at 2000g for 1 min, resuspended and equally divided into four fresh 1.5 ml tubes. Aliquoted beads were pelleted and drained of supernatant. Drained beads were suspended and rotated in 1 ml of pH-specific buffers for 55–60 min (150 mM NaCl, 10 mM MgCl_2_, 10% glycerol, HALT protease inhibitor, 0.5% NP40 in 50 mM MES pH 5.6 / pH 6.1 / 50 mM HEPES pH 7.0 / 25 mM HEPES, 25 mM MOPS pH 7.4). Beads were pelleted, washed twice with the same pH-specific buffer and twice with pH 7.0 buffer. The last buffer was completely removed from the beads, and complexes were eluted with 2 x NuPAGE LDS sample buffer with 100 mM DTT, boiled for 5 min at 95°C. Samples were analysed by Western blotting as above. Western blots were quantified (BioRad ImageLab 6.0.1) and analysed in GraphPad Prism.

#### 4.8.3. Ph-dependent immunoprecipitation (GFP-Caprin-1)

20 × 10^6^ U2OS ΔCaprin-1 cells stably expressing GFP-Caprin-1-WT or GFP-Caprin-1-FGDF chimera were lysed in 1.5 ml (50 mM HEPES, 150 mM NaCl, 2.5 mM EGTA, 0.5% NP40, 10% glycerol, 1 mM DTT, HALT protease inhibitors (Thermo Fisher), 50 µg ml^−1^ RNase A (NEB). Lysates were rotated for 10 min at 4°C, sonicated for 8 min on ice, cleared by centrifugation (10 000 g, 10 min, 4°C) and incubated with GFP-Trap agarose beads (ChromoTek) for 1–2 h with continuous rotation at 4°C. Then, beads were processed as described above for pH-dependent IP (G3BP1).

### 4.9. Immunofluorescence analysis

#### 4.9.1. High-content microscopy

U2OS ΔΔG3BP1/2 cells stably expressing GFP-G3BP1-WT or mutants and U2OS ΔCaprin-1 cells stably expressing GFP-Caprin-1-WT or FGDF chimera were fixed and processed for fluorescence microscopy as described previously [95]. Briefly, cells were plated on a µ-Plate 24 Well Black plates ibiTreat (IBIDI), left unstressed or stressed with 200 µM sodium arsenite for 1 h, fixed using 3.7% formaldehyde (Sigma) in PBS for 10 min and followed by 5 min post-fixation/permeabilization in ice-cold methanol. Cells were blocked for 1 h in 5% horse serum/PBS, and primary and secondary incubations were performed in blocking buffer for 1 h. All secondary antibodies were raised in donkey against either mouse, rabbit or goat and tagged with either Alexa Fluor 488, 568 or 647 (Thermo Fisher). Following washes with PBS, fixed and stained cells were kept in PBS. Images were recorded with a Molecular Devices ImageXpress Micro microscope, equipped with a 20 x or 40 x objective and illuminated with a mercury lamp and standard filters for DAPI, FITC, Cy3 and Cy5. Images were captured using a four-megapixel sCMOS digital camera with the manufacturer's software MetaXpress, and raw TIF files were analysed using CellProfiler (CP), ImageJ and Rstudio [81,89,96–98]. Our CP pipelines identified cellular outlines in the GFP channel by applying a propagation algorithm initialized from DAPI-stained nuclear regions. The nuclear region was subtracted from cellular outlines to define cytoplasm objects. Median filter smoothing and speckle-enhancing modules were applied to enhance granule signals that were identified separately in both channels as primary objects in both green and red channels. Identified granules were related to each other as well as to their parent cytoplasm objects for relational analysis. Several parameters such as granule area and cytoplasmic mean GFP fluorescence intensity were exported as tables for data tidying, analysis and visualization in *R*. The fluorescence mean intensity values obtained from CP were range-normalized to set the median value of all cells from an entire dataset to one (*F*_rel_) (electronic supplementary material figures 7C and 8C). Cells were sorted digitally into bins with a width of 0.1 *F*_rel_-units for each construct and experiment. Bins comprising *n* < 10 cells were not analysed. Apparent DR curves were obtained by plotting the median SG area of each bin against its respective *F*_rel_-value and analysed applying the general asymmetric five-parameter logistic model as implemented in the drc package [99] (electronic supplementary material, figure S7D). All datasets within a single channel were normalized to the respective fitted maximal response of the wild-type DR curve for final analysis (figure 5*b* and 7*d*; electronic supplementary material, figure 8E).

#### 4.9.2. Ratiometric pHluorin2-G3BP1-WT measurement

Cells were plated on µ-Slide 8 Well ibiTreat chamber slide (IBIDI) and grown for an additional 24 h. Cells were then left untreated or treated with 200 µM sodium arsenite diluted in complete media or 20 µM clotrimazole diluted in serum-free medium for 30 min then viewed with a Supercontinuum Confocal Leica TCS SP5 X, equipped with a pulsed white light laser, 405 nm violet diode laser, a Leica HCX PL Apo 63x/1.40 oil objective and a heated chamber set on 37°C. Live cell images were recorded sequentially with the following settings to avoid saturated signals. Pinhole was set to 2 airy. First: excitation at 485 nm with 25% intensity of the white laser light, PMT1 (photomultiplier tube) detector was set to gain 900 V and offset −1.0 V, emission window was set from 505 to 515 nm. Second: excitation at 405 nm of the violet diode laser with 25% intensity, PMT1 detector was set to gain 900 V and offset −1.0 V, emission window was set from 505–515 nm. Quantification was performed on four images with 3–4 cells and multiple SGs per cell, which totalled around 40 or more SGs per experiment. The area of the region of interest (ROI) was set equal in SGs and in adjacent cytoplasm and analysed. Then, the mean pixel values of channel 485 nm and 405 nm were measured, and the 405/485 nm ratio was calculated and plotted [58].

#### 4.9.3. Full-length recombinant G3BP1 production

Full-length recombinant G3BP1-WT, H31A and H31YH62Y proteins were expressed in T7 Express *lysY/I^q^* competent *E. coli* cells at 25°C overnight in Terrific broth. Bacterial cells were resuspended in 120 ml lysis buffer (50 mM Tris pH 8.0, 300 mM NaCl, 5% glycerol, 0,1% TritonX100, 15 mM Imidazole, protease inhibitor (Pierce), nuclease (500 units, Pierce), 12 µL RNAse (20 mg ml^−1^, NEB)) and disrupted by sonication. Cleared lysates were manually applied to HisTrap FF 5 ml (Merck) columns and firstly washed with 20 mM imidazole, 50 mM Tris pH 8.0, 300 mM NaCl, 5% glycerol, then with 20 mM imidazole, 50 mM Tris pH 8.0, 1000 mM NaCl, 5% glycerol and finally with 40 mM imidazole, 50 mM Tris pH 8.0, 300 mM NaCl, 5% glycerol. Bound G3BP1 proteins were eluted in 50 mM HEPES pH 7.4, 400 mM NaCl, 1 mM DTT, 500 mM imidazole. Eluted proteins were analysed by SDS-PAGE and stained with Coomassie blue. Then, purified G3BP1 proteins were digested with TEV protease (200 µg) in elution buffer with additional EDTA (0.5 mM) and then incubated overnight at 4°C, rocking. Tag-removed G3BP1 was concentrated and directly loaded onto HiTrap Heparin HP affinity column (Cytiva) in order to remove non-specific RNA binding (Buffer pairs are 20 mM MES pH 6.0, 5% glycerol and 20 mM MES pH 6.0, 1.5 M NaCl, 5% glycerol). Fractions with target protein were then concentrated and changed to buffer 50 mM HEPES pH 7.4, 400 mM NaCl, 1 mM DTT by using PD10 desalting column. A small fraction of the purified G3BP1 was applied onto a HiLoad 16/600 Superdex 200 prep grade column (GE Healthcare) for checking the polymerization status of G3BP1. The final concentrated G3BP1 was concentrated to around 12 mg ml^−1^ and stored at −80°C.

#### 4.9.4. *In vitro* reconstituted condensate assays

Condensates were reconstituted following established protocols [57]. In brief, U2OS ΔΔG3BP1/2 cells stably expressing GFP-G3BP1-WT, H31A, H31YH62Y or U2OS ΔCaprin-1 cells stably expressing GFP-Caprin-1-WT or FGDF chimera were trypsinized, washed, counted and collected in a 50 ml tube. 1 × 10^7^ cells were lysed for 3 min at RT in 250 µl lysis buffer (50 mM HEPES, 0.5% NP40, protease inhibitor and 2.5% murine RNase inhibitor (NEB)), spun down for 5 min at 21 000 g at 20°C. For assays with varied pH values, 50 µl of lysates obtained at pH 7.4 with modified lysis buffer (25 mM HEPES, 25 mM MES, 25 mM sodium acetate, 0.5% NP40, protease inhibitor and 2.5% murine RNase inhibitor (NEB)) were adjusted to obtain final pH values between 6.8 and 7.8. Total protein concentration was adjusted to 5 mg ml^−1^. For LLPS induction rG3BP1-WT, H31A, H31YH62Y were diluted in 50 mM HEPES pH 7.4, 400 mM NaCl, 1 mM DTT to reach final concentrations of 180, 150, 120, 90, 60 and 30 µM. Then 5 µl of rG3BP1 proteins were added to 25 µl of GFP-G3BP1 or GFP-Caprin-1 lysates and mixed. Twenty-five microlitres of the mixture was immediately transferred to IBIDI 18-well microscope chamber slides and incubated for 1 h at RT. Images were taken with a Supercontinuum Confocal Leica TCS SP5 X, equipped with a pulsed white light laser and a Leica HCX PL Apo 63x/1.40 oil objective. Condensates were analysed with CellProfiler.

To determine the pH standard curve of *in vitro* reconstituted condensates, ΔΔG3BP1/2 U2OS cells stably expressing pHluorin2-G3BP1-WT or pHluorin2 were used for *in vitro* reconstitution as described above. Images were taken with a Zeiss LSM Airy 980 with an 63 x oil objective and recorded sequentially with the following settings. First: laser 488 nm, GaAsP-PMT detector (499–525 nm), gain 850 V, offset 0 V. Second: laser 405 nm GaAsP-PMT detector (499–525 nm), gain 850 V, offset 0 V. Image resolution is 1024 × 1024 in 16 bit. Quantification of condensates and their adjacent area was performed on four independent biological experiments with five images taken and 10 condensates per image analysed, which totalled 50 or more condensates per experiment. The area of the ROI was set equal in condensates and in adjacent areas and analysed. Then, the mean intensity values of channels 488 nm and 405 nm were measured and 405 and 488 nm ratio was calculated and plotted [58].

## Data Availability

Supplementary data are made publicly available from the Dryad Digital Repository https://doi.org/10.5061/dryad.k98sf7mb8 [100] and https://github.com/derpaule/RSOB-22-0369. The crystal structure was deposited with PDB-ID 6TA7. The data are provided in the electronic supplementary material [101].
